# Moral injury prevention and intervention

**DOI:** 10.1080/20008066.2025.2567721

**Published:** 2025-10-21

**Authors:** Victoria Williamson, Radha Kothari, Amanda Bonson, Gavin Campbell, Neil Greenberg, Dominic Murphy, Danielle Lamb

**Affiliations:** a Institute of Psychology, Psychiatry and Neuroscience, King's College London, London, UK; b University of Bath, Bath, UK; c King's College Hospital, NHS Trust, London, UK; d Centre for Applied Military Health Research, Combat Stress, Leatherhead, UK; e Department of Applied Health Research, NIHR ARC North Thames, UCL, London, UK

**Keywords:** Moral injury, intervention, prevention, trauma, occupational, wellbeing, Daño moral, prevención, trauma, intervención

## Abstract

**Background:** Those working in high-risk occupations may often face ethical dilemmas that violate their moral code which can lead to moral injury (MI). While research into the impact of MI is growing, evidence for effective treatment interventions and prevention approaches remains limited.

**Objective:** To review recent developments in treatment and prevention approaches for MI-related mental health difficulties.

**Method**: We synthesised emerging treatments, including trauma focused therapies and spiritual approaches, as well as possible prevention strategies.

**Results:** Conventional treatments for post-traumatic stress disorder (PTSD) (e.g. prolonged exposure) often inadequately address MI and may exacerbate symptoms. Adapted or novel approaches, including Impact of Killing, Adaptive Disclosure, and Restore and Rebuild, show promise, particularly when co-produced with patients and clinicians. Spiritual interventions demonstrate mixed outcomes. Prevention research remains very limited but highlights the potential of systemic reforms, leadership fostering psychological safety, preparedness training, structured reflection, and peer support. Evidence remains constrained by small samples, military-focused populations, and inconsistent measurement of MI.

**Conclusions:** While no gold-standard intervention exists, values-based and compassion-focused approaches appear promising. Prevention strategies targeting organisational culture and fostering preparedness are urgently needed, particularly for civilian and diverse occupational groups, to better support and protect those exposed to potentially morally injurious events.

## HIGHLIGHTS

- Moral injury (MI) occurs when potentially morally injurious events (PMIEs) violate an individual’s moral code, leading to intense guilt, shame, and anger. Strongly associated with PTSD, depression, and suicidality, MI is increasingly recognised beyond military contexts, affecting first responders, healthcare, and media workers, with significant consequences for psychological wellbeing and occupational functioning.
- Standard PTSD treatments often fail to address MI-specific symptoms and may worsen guilt or shame. Emerging approaches such as Adaptive Disclosure, Impact of Killing, and Restore and Rebuild show promise, especially when co-produced with patients. These therapies emphasise values-based behaviour, self-compassion, and moral repair, but evidence remains limited to small, predominantly military-focused studies.
- Prevention research is extremely limited. Leadership that fosters psychological safety, preparedness training, structured reflection, and peer support may reduce risk of MI. Systemic reforms – such as improved working conditions and fairer workloads – are also recommended.

## Abstract (es)

**Antecedentes:** Aquellos que trabajan en ocupaciones de alto riesgo pueden con frecuencia enfrentarse a dilemas éticos que violan sus códigos morales lo que puede conducir al daño moral (DM). Aunque la investigación acerca del impacto del DM va en aumento, la evidencia para intervenciones de tratamiento y enfoques de prevención eficaces aún son limitadas.

**Objetivo:** Revisar los desarrollos recientes en enfoques de tratamiento y prevención para las dificultades de salud mental relacionadas con DM.

**Método:** Se sintetizaron los tratamientos emergentes, incluidas terapias centradas en trauma y enfoques espirituales, así como posibles estrategias de prevención.

**Resultados:** Los tratamientos convencionales para el trastorno de estrés postraumático (TEPT) (tales como exposición prolongada) con frecuencia abordan el DM en forma inadecuada y pueden exacerbar los síntomas. Los enfoque adaptados o novedosos, incluido el Impacto de Matar, la Revelación Adaptativa y la Restauración y Reconstrucción, son promisorios, particularmente cuando se co-producen con pacientes y profesionales clínicos. Las intervenciones espirituales han mostrado resultados mixtos. La investigación en prevención es aun muy limitada, pero destaca el potencial de las reformas sistémicas, el liderazgo que fomenta la seguridad psicológica, la capacitación en preparación, la reflexión estructurada y el apoyo entre pares. La evidencia sigue limitada por el tamaño reducido de las muestras, poblaciones centradas en el ámbito militar y la medición inconsistente del DM.

**Conclusiones:** Si bien no existe una intervención gold-standard, los enfoques basados en valores y centrados en la compasión parecen prometedores. Se necesitan con urgencia estrategias de prevención que fomenten la cultura organizacional y la preparación, particularmente para grupos civiles y ocupacionales diversos, para brindar un mejor apoyo y protección a quienes se exponen a eventos potencialmente perjudiciales para la moral.

## 1. Introduction

Individuals working in high-risk occupations – such as first responders, military personnel, and media workers  – will often face moral dilemmas during their careers. Many carry the heavy burden of the choices they made, or failed to make, long after the moment has passed. For some individuals, experiencing events that violate their moral code can lead to the development of moral injury (MI).

MI is the intense distress experienced after events that violate one’s moral code (Bonson et al., 2023). Potentially morally injurious events (PMIEs) that can lead to MI are categorised as acts of commission, omission or betrayal. An example of an act of commission can include where a soldier follows an order to fire at an enemy combatant and civilian bystanders are killed; or a veterinary surgeon who euthanises an otherwise healthy animal who can no longer compete. An example of an act of omission can include a social worker who has concerns that a child could be experiencing abuse, but due to a lack of foster care places, they do not recommend the child’s removal from the home and the child later becomes badly injured. A betrayal PMIE can include a female police officer who experiences a sexual assault by a colleague; the female officer reports the assault to their chain command who does not thoroughly investigate her complaint, and she is branded as ‘difficult’ to work with.

Research suggests that MI is characterised by strong feelings of guilt, shame, and anger (Griffin et al., 2019; Litz et al., 2009; Williamson et al., 2021). Theoretical models suggest that these difficulties can persist over time because disrupted meaning-making, self-condemnation, and loss of trust impede emotional processing and integration, thereby sustaining psychological distress (Atuel et al., 2021; Bonson et al., 2023; Litz et al., 2009). While MI is not a diagnosable mental disorder, studies have shown a strong association between MI and PTSD, depression, and suicidality (Griffin et al., 2019; Griffin et al., 2025; Williamson et al., 2018). Studies find that clinicians report uncertainty in treating MI-related mental disorders due to a lack of validated treatments and limited accessible training (Williamson et al., 2021; Williamson et al. (2021). PTSD treatments, such as prolonged exposure (PE) or trauma focused cognitive behavioural therapy (TF-CBT) may not adequately address MI-related mental health disorders. Patients with MI report existing treatments do not fully address their symptoms (Bonson et al., 2024). International studies argue that treatments, such as PE, may exacerbate symptoms of guilt and shame (Maguen & Burkman, 2013). Other treatments, such as TF-CBT which aim to update inaccurate/maladaptive appraisals, may not be suitable for cases of MI where distress stems from PMIEs where appraisals of blame may be justified or appropriate (Steinmetz & Gray, 2015).

## 2. What treatments exist for MI-related mental health disorders?

Efforts have been made to develop novel treatments for MI-related mental disorders. A rapid review (Varker et al., 2022) of MI treatments published between 2015–2022 found considerable uncertainty of the best treatment approach as the methodological quality of many studies was poor. In addition to this, several other reviews have examined both the construct of MI and interventions designed to address MI-related mental health difficulties (Griffin et al., 2019; Harris et al., 2021). While this article is not an exhaustive review, we summarise several of the more recently published approaches and their outcomes (see Table 1).

**Table 1.** Description of interventions to address moral injury-related mental disorders.

Author	Country	Intervention	*N*	Sample population	No.	Mode of delivery	Setting	Provider	Treatment targets	Findings
Griffin et al., 2015	USA	Workbook	102	University students	6^b^	Workbook	Individual	N/A	Workbook intended to promote self-forgiveness focusing on repairing social bonds, reaffirmation of values and restoring positive self-regard and reducing rumination.	Increases in self-forgiveness and decreases in self-condemnation among perpetrators of interpersonal wrongdoing post-tx. Greater perceived transgression severity predicted stronger improvements in self-forgiveness, guilt, and shame.
Kelley et al., 2025	USA	MMMI	28	Military veterans	7	Online	Group	Clinician	Information on mindfulness, application of mindfulness in daily life, discussion of MI, strengthening compassion towards self and others.	Reduction in MI symptoms in MMI group but whether change is statistically significant is unclear. Drop out unclear.
Litz et al., 2024	USA	AD-E	89	Military veterans	12	In-person	Individual	Clinician	AD adapted to include loving kindness/compassion meditation, mindfulness training.	Significant reduction in AD-E group PTSD, anger and functional impairment post-tx. No significant reduction in alcohol misuse.
Litz et al., 2021	USA	AD	62	Marines/Sailors	8	In-person	Individual	Clinician	Imaginal exposure, imaginal dialogue with forgiving moral authority, discussion of event and positive reframing of meaning of event.	Significant reduction in AD group PTSD symptoms post-tx. Patients who received AD responded similarly to those who received CPT.
Maguen et al., 2017	USA	IOK	17	Military veterans	6–8	In-person	Individual	Clinician	Cognitive behavioural treatment focused on self-forgiveness, improving self-compassion, fostering values-based behaviours and amends making.	Post-tx improvements in IOK group PTSD and psychological distress. IOK group improvements in post-tx community reintegration were not statistically different from control.
Norman et al., 2022	USA	TrIGR	74	Military veterans	6–7	In-person	Individual	Clinician	Cognitive behavioural intervention focusing on reducing shame and guilt by discussing guilt source, cognitive restructuring by identifying value-based function guilt serves, and promoting adaptive values-based goals.	Significant reduction in TrIGR group PTSD, depression, shame and psychological distress post-tx. Quality of life unchanged post-tx.
Smith-MacDonald et al., 2023	Canada	3MDR	11	Military personnel/veterans	6	In-person	Individual	Clinician	Exposure based trauma therapy delivered using VR and physical movement, includes exposure to traumatic materials, awareness and expression of emotion, reconsolidating the trauma memory via dual attention task.	Unclear whether 3MDR treatment leads to significant reduction in symptoms. Drop out reportedly due to CV19 restrictions.
Walser et al., 2024	USA	ACT-MI	16	Military veterans	6–8	In-person	Group	Clinician	Acceptance and mindfulness techniques, acceptance of moral pain, clarification of values, reconnecting to values-based behaviours.	Increase in value-aligned behaviour post-tx. Reduction in post-tx PTSD and depression symptoms but unclear if change statistically significant. Patients received other treatments concurrently during this trial.
Williamson, Murphy, et al., 2023	UK	R&R	20	Military veterans	20	Online	Individual	Clinician	Processing the event(s), improving self-compassion, strengthening connections with others, and reconnecting with core values.	Significant reduction in PTSD, depression, alcohol misuse and MI-related distress symptoms post-tx.
*Spiritual interventions*										
Bormann et al., 2012	USA	ESWB	66	Military veterans	6	In-person	Group	Unclear	Mantra repetition mediation as adjunct to care as usual.	Significant reduction in ESWB group PTSD symptoms, increase in spiritual wellbeing post-tx. Drop out unclear.
Harris et al., 2018	USA	BSS	71	Military veterans	8	In-person	Group	Chaplain	Spiritually integrated counselling aiming to resolve spiritual distress by addressing relationship with a Higher Power, promoting adaptive meaning making of traumatic events, and increased use of active coping strategies.	Both BSS and PCGT significantly reduced PTSD symptoms post-tx, but BSS was more effective for distress related to a Higher Power.
Smigelsky et al., 2022	USA	REAL	15	Military veterans	12	In-person	Group	Chaplain & clinician	Making sense of loss, sharing distress as a group, reimagine life from new perspective.	Reduction in PTSD and depression post-tx but unclear if statistically significant change.
Starnino et al., 2019	USA	SFM	24	Military veterans	8	In-person	Group	Chaplain & clinician	Focus on resolution of anger, forgiveness, meaning making and rebuilding a spiritual foundation.	Significant post-tx reduction in PTSD and modest reduction in spiritual injury symptoms. No significant change in positive religious coping post-tx. Drop out unclear.

NOTE. MI = moral injury. N = number of trial patients who receive MI intervention. No. = number of MI intervention treatment sessions. Post-tx = post-treatment. BL = baseline. PTSD = posttraumatic stress disorder. 6^b^ = six-hour self-directed workbook intervention. CPT = cognitive processing therapy. CV19 = COVID 19. ACT-MI = Acceptance and Commitment Therapy for Moral Injury (Walser et al., 2024). 3MDR = Multi-modal Motion-Assisted Memory Desensitization and Reconsolidation (Smith-MacDonald et al., 2023). VR = virtual reality. MMMI = Mindfulness to Manage Moral Injury (Kelley et al., 2025). R&R = Restore and Rebuild (Williamson, Murphy, et al., 2023). TrIGR = Trauma Informed Guilt Reduction (Norman et al., 2022). AD-E = Adaptive Disclosure Enhanced (Litz et al., 2024). PE = prolonged exposure. AD = Adaptive Disclosure (Litz et al., 2017). IOK = Impact of Killing (Maguen et al., 2017). Workbook = Self Forgiveness Workbook (Griffin et al., 2015). BSS = Building Spiritual Strengths (Harris et al., 2018). PCGT = Present Centered Group Therapy. REAL = Reclaiming Experiences and Loss (Smigelsky et al., 2022). ESWB = Existential Spiritual Wellbeing (Bormann et al., 2012). SFM = Search for Meaning (Starnino et al., 2019).

Treatments evaluated for MI-related mental health disorders have included standard trauma-focused treatments, such as Cognitive Processing Therapy (CPT) (Held et al., 2020; Litz et al., 2017), as well as adapted versions of therapies such as Modular Motion-Assisted Memory Desensitisation and Reconsolidation (3MDR) and Acceptance and Commitment Therapy (ACT) (Aita et al., 2023; Litz et al., 2021; Varker et al., 2022). Novel promising treatments have also been developed, including Restore and Rebuild (R&R), Adaptive Disclosure (AD), and Impact of Killing (IOK) (Litz et al., 2021; Maguen et al., 2017; Williamson, Murphy, et al., 2023). Many of the novel targeted treatments aim to foster emotional regulation and values-driven behaviour, and non-judgementally examine patients’ experiences of the PMIE, compassionately exploring negative appraisals (Table 1). It is argued that as a patient’s symptoms of guilt and shame reduce in treatment, so does their use of avoidant coping strategies meaning patients are more able to engage in value-driven activities and this contributes to symptom reduction (Held et al., 2020). Co-produced therapies, such as IOK and R&R, which included the views of patients and clinicians in treatment development report high levels of acceptability and positive patient outcomes maintained at follow up (Purcell et al., 2018; Williamson, Murphy, et al., 2023). However, it is difficult to draw firm conclusions about the overall effectiveness of many of these treatments as most evidence is drawn from case studies, trials with small samples, the inclusion of primarily military and male populations, and problematic measurement of MI (Aita et al., 2023; Varker et al., 2022) As few studies report time since PMIE or exposure type (e.g. betrayal, omission, commission), it is unclear when particular treatments may be most effective, for which patients, and in what circumstances.

Interventions which focus on addressing patient’s spiritual beliefs (e.g. Building Spiritual Strengths [BSS]; Search for Meaning [SFM]; Starnino et al., 2019; Winkeljohn Black & Klinger, 2022) have also been developed, which can be delivered by chaplains or clinicians. Such spiritual-focused interventions aim to improve MI-related symptoms (e.g. alienation from a higher power), foster the use of spiritual strategies, and reengage with social connections. However, whether these spiritual interventions are effective is unclear as significant improvements in patient symptoms post-treatment have not been consistently found or maintained at follow up (Harris et al., 2018; Starnino et al., 2019). This requires further investigation as studies have found an inconsistent relationship between religious coping practices and psychological adjustment post-trauma (Koenig et al., 2018). Whether these spiritual interventions will be applicable to those with no extant spiritual faith is also unclear and may limit their scalability and generalisability.

## 3. What prevention interventions exist for MI-related mental health disorders?

Moral distress following PMIEs is arguably a natural response to events which violate one’s moral code (Farnsworth et al., 2017) and can be a catalyst for right action (e.g. stepping up and advocating for a mistreated patient). When moral distress goes unaddressed and becomes chronic and severe, accompanied by enduring negative changes in beliefs about oneself and others, MI can develop and contribute towards significant mental health difficulties (Bonson et al., 2023). Preventing PMIEs exposure may not always be feasible, given the enduring realities of war, human suffering, injustice and the necessities of particular occupations. However, timely support and intervention may prevent moral distress from escalating to MI.

With no clear gold-standard MI treatment on the horizon, there is a need for prevention interventions to protect and support staff working in high-risk occupations where PMIEs are common. Despite the large number of studies exploring the prevalence and impact of MI in various populations (Griffin et al., 2019); Griffin et al., 2023), research into MI prevention remains in its infancy. Research studies aiming to co-design prevention interventions are underway (Williamson et al., in press), with further evaluations needed.

### 3.1. Systemic prevention approaches

Steps to prevent MI have been recommended at organisation, team and individual levels. From an organisation or systemic perspective, improving organisational working conditions, increasing staffing levels to better manage and fairly distribute workloads, setting more reasonable performance demands, and enhancing compensation could improve morale and reduce the likelihood of some PMIEs occurring (Dennard et al., 2021; Reamer, 2022). Whether this is feasible in resource limited settings (e.g. healthcare, prisons) is beyond the scope of this article. However, the potential cost of inaction could be significant. Staff with MI report leaving or considering leaving their roles (Stanojević & Čartolovni, 2022; Williamson et al., 2023). As rates of staff turnover, absenteeism, and burnout continue to rise particularly in underfunded public sector contexts, the result – if this trend remains unaddressed – will likely be a more stressed workforce facing poorer working conditions and potentially encountering a greater number of PMIEs. Economic evaluations of the cost of MI could provide evidence to support the need for prevention interventions.

### 3.2. Fostering preparedness

To mitigate against organisational and funding constraints, prevention efforts that can be implemented in the short-term must be scalable, practical and cost-effective. Feeling unprepared for one’s role is a risk factor for MI (Williamson, Murphy et al., 2020). Having frank and open conversations as a team, led by those in senior roles or management, about the potential for PMIEs in the role as well as the psychological responses that could be experienced as a result, has been recommended to foster psychological preparedness and safeguard against MI development (Williamson et al., 2020). Previous studies have found that professionals affected by MI (Agazio & Padden, 2024; Williamson, Murphy et al., 2020), cope by framing their actions during the PMIE as efforts to ‘do the right thing’ or ‘for the greater good.’ Preparedness training which acknowledges that rules of engagement may not always be clear, the inevitability of ethical grey areas, and promotes ethical and values-based (e.g. respect, integrity, courage) decision making can help normalise the challenges that professionals may face, support coping and decrease the likelihood of unethical decisions (Troy, 2024). This preparedness training should also incorporate discussion of PMIEs that one should not expect to be exposed to (e.g. workplace harassment, bullying) and what recourse to take should these occur. Recent recommendations for approaches to frankly prepare staff for possible PMIE exposure have included training via virtual reality simulations of ethically challenging scenarios which may trigger moral distress (Sivanathan et al., 2022; Martin et al., 2024) – however, the effectiveness of such training remains uncertain as trials are still underway.

### 3.3. Prevention via strong leadership

Previous studies have found inclusive leadership which fosters psychological safety, mutual respect and trust can moderate the psychological impact of workplace bullying on staff suffering with MI (Srivastava et al., 2024). It has also been argued that those in leadership should regularly and proactively ‘check in’ with their team, offering mentoring, and empathetic support (Williamson et al., 2020). Providing managers with training about MI and the early warning signs of staff distress (Greenberg & Tracy, 2020) may increase their sensitivity to and confidence in discussing MI with their teams.

Creating suitable settings where PMIEs can be openly discussed and understood, including identifying and implementing measures to prevent event reoccurrence, is argued to be essential for facilitating moral repair (Shale, 2020). An organisational culture which promotes psychological safety where vulnerability is normalised, mistakes are acknowledged and approached as learning opportunities with a mindset of non-judgementally listening to other points of view may relieve some of the moral burden professionals face. Organisations that claim to have ‘learnt’ from events while not effectively implementing recommendations may cause more harm and be seen as further betrayal. This is especially concerning given recent findings suggesting that betrayal-based PMIEs are among the most prevalent (Ter Heide & Olff, 2023; Williamson, Lamb, et al., 2023). As MI can erode trust in others (Bonson et al., 2023), it is also crucial that those in leadership ensure there are fair, confidential and robust internal complaints procedures which protect staff from fears of interpersonal conflict or retaliation when reporting incidents. Promoting open PMIE reporting over silence, withdrawal or resignation may protect against MI (Srivastava et al., 2024).

Strategies for fostering staff wellbeing more broadly include recommendations that those in leadership encourage and model good work-life balance, celebrate staff achievements, ensure staff workloads are manageable, champion and implement wellbeing training, and signpost evidence-based formal support (Dennard et al., 2021). Many of these suggestions are likely to be helpful in fostering atmospheres where, while PMIEs may occur, staff are better supported and able to respond adaptively.

### 3.4. Shared decision making, team collaboration and peer-support

To prevent MI in healthcare workers during the COVID-19 pandemic at a team level, it was recommended that healthcare teams hold regular, well-documented interdisciplinary meetings to make clinical decisions collaboratively. This approach, with input from senior clinicians – particularly for less experienced clinicians  – was expected to help mitigate any negative psychological effects on staff if poor patient outcomes occurred (Williamson, Greenberg et al., 2020). Working in small teams that promote a sense of unified purpose, belonging, and shared goals, was also recommended, as it could allow for at-risk staff members to be identified earlier and foster peer support (Williamson, Greenberg et al., 2020). There is good evidence that social support is beneficial post-trauma and encouraging staff who frequently encounter PMIEs to seek social support within their teams, from trusted loved ones or support from other avenues (e.g. chaplaincy, helplines, etc) has been advocated (Williamson et al., 2020). Social support from peers that provides space for those exposed to PMIEs to discuss the PMIE itself may normalise emotional responses to distressing events, reducing feelings of isolation as well as support professionals in navigating future challenging situations (DiCiro et al., 2024)

### 3.5. Potential post-event interventions

Many interventions, such as Critical Incident Stress Debriefing (Mitchell & Everly, 1995) or Psychological First Aid (Figueroa et al., 2022), have been evaluated for PTSD prevention in professionals frequently exposed to trauma. A recent review (Billings et al., 2023) found no clear advantages of any specific intervention, or evidence of one being superior to another; however, it did note that generic debriefing was associated with negative outcomes. Whether these interventions are protective against MI remains unknown. Nonetheless, a common thread was that post-incident interventions were more acceptable when delivered in group settings by someone viewed as credible and familiar with the specific demands of the employees’ work (Billings et al., 2023) – an insight that may help guide future MI prevention approaches.

Reflective practice where staff regularly come together to reflect on and discuss the rewards and emotional demands of their work has also been suggested as potentially protective against the development of MI (Hegarty et al., 2022). While the emotional benefits of reflective practice have been reported, organisations will need strategies in place to overcome practical hurdles to bringing staff together who may be in understaffed teams, working in shifts, have limited meeting spaces, or have stigma-related concerns about making emotional disclosures in a group setting. It is notable that trials investigating the impact of staff training – comprising of education (e.g. theories of ethical decision making) and facilitated discussions of ethically challenging dilemmas staff have encountered – found no significant reduction in moral distress amongst participants compared to those who did not receive training (Sporrong et al., 2007).

Several promising interventions for MI-related mental health difficulties (see Table 1) include sessions that focus on non-judgementally exploring the event while gently challenging patient appraisals of shame and guilt by examining what factors were within one’s power and control at the time. Interventions also include sessions aiming to increase patient self-compassion (e.g. loving kindness meditation (Gilbert, 2014) and sessions which explore and encourage values-driven behaviours. It is possible that prevention interventions which incorporate these dimensions may be beneficial. Prevention interventions will also need to consider the context in which support is provided. The effectiveness of psychological interventions remains uncertain when individuals are exposed to ongoing trauma (Ennis et al., 2021). For example, a prison officer experiencing betrayal-based MI difficulties from repeated workplace harassment, or a soldier struggling with omission-based MI due to rules of engagement on deployment, may rely on cognitive avoidance and suppression as strategies to survive their environment (Ehlers & Clark, 2000). Effective MI-related approaches in these circumstances must prioritise individuals’ safety and should ideally be implemented when the risk of the MI being reinforced is not ongoing.

### 3.6. Psychoeducation

At an individual level, recommendations to prevent MI have included incorporating MI-related psychoeducation into existing wellbeing educations. A recent review found some evidence that psychoeducation can improve knowledge of and attitudes towards mental health, but no support for the routine use of psychoeducation as a stand-alone approach to prevent mental health problems post-trauma (Brooks et al., 2021). Therefore, psychoeducation as a stand-alone prevention intervention for MI is unlikely to be sufficient.

Providing opportunities for staff to learn about and practice adaptive coping strategies which have a holistic focus, including exercise, nutrition, emotional regulation, and cognitive strategies (e.g. positive reappraisal) (Canning et al., 2025) may also be beneficial, as staff struggling with MI often report poor physical health and use maladaptive coping strategies to manage their distress (Williamson, Murphy, et al., 2020). Central to the success of such education initiatives is likely to be their endorsement by leadership, with managers ensuring staff have opportunities to attend.

## 4. Conclusions

Research into the experience and impact of MI has grown exponentially. What began as an examination of the phenomena in military veterans has expanded to include many high-risk populations. While indecision over the correct classification of PMIEs and the precise definition of MI persists in academic and theological circles, what can be agreed is that the fallible nature of humanity – our collective capacity for wrong action, errors and mistakes even with the best of intentions – means that PMIEs do occur and can have lasting negative effects. Research has improved our responses to MI-related mental health difficulties, and every trial brings us closer to being able to offer a validated, effective treatment that targets patients intense psychological, interpersonal and existential struggle. Interventions that promote values-based behaviours and support the re-evaluation of perceived responsibility through a self-compassionate lens may help individuals who feel they made the wrong choice – or feel they were harmed by the wrong choices of others  – to heal. Further research is needed, particularly in civilian and non-Western populations, to clarify which interventions are most effective, for whom, and in response to which types of experiences. Advancements in MI measurement tools and greater attention to the physical health consequences of MI will also be essential to strengthen the evidence base.

As awareness of the impact of MI grows, the lack of research focused on prevention becomes harder to overlook. We owe it to the professionals whose roles expose them to moral dilemmas to shift our focus towards a proactive approach that better equips them with the tools and support needed to prevent moral distress from developing into injury. Responsibility for addressing the systemic conditions which contribute towards workplace PMIEs, such as under-staffing and poor working conditions, lies not only with individual organisations but also with policy makers responsible for public sector funding. Preventative efforts might begin with developing training that fosters preparedness, MI psychoeducation, and holistic wellbeing initiatives. Encouraging transformational leadership, creating safe avenues for reporting PMIEs, alongside structured opportunities for reflection and discussion, may help individuals process PMIEs and navigate them more effectively in the future. Preventing MI is undoubtably a complex challenge, but it also presents an opportunity to create an innovative multidisciplinary approach to provide professionals with the resources and support they need, ultimately promoting better long-term mental health and sustaining their ability to remain well and in work.

## Data sharing

No data available.
